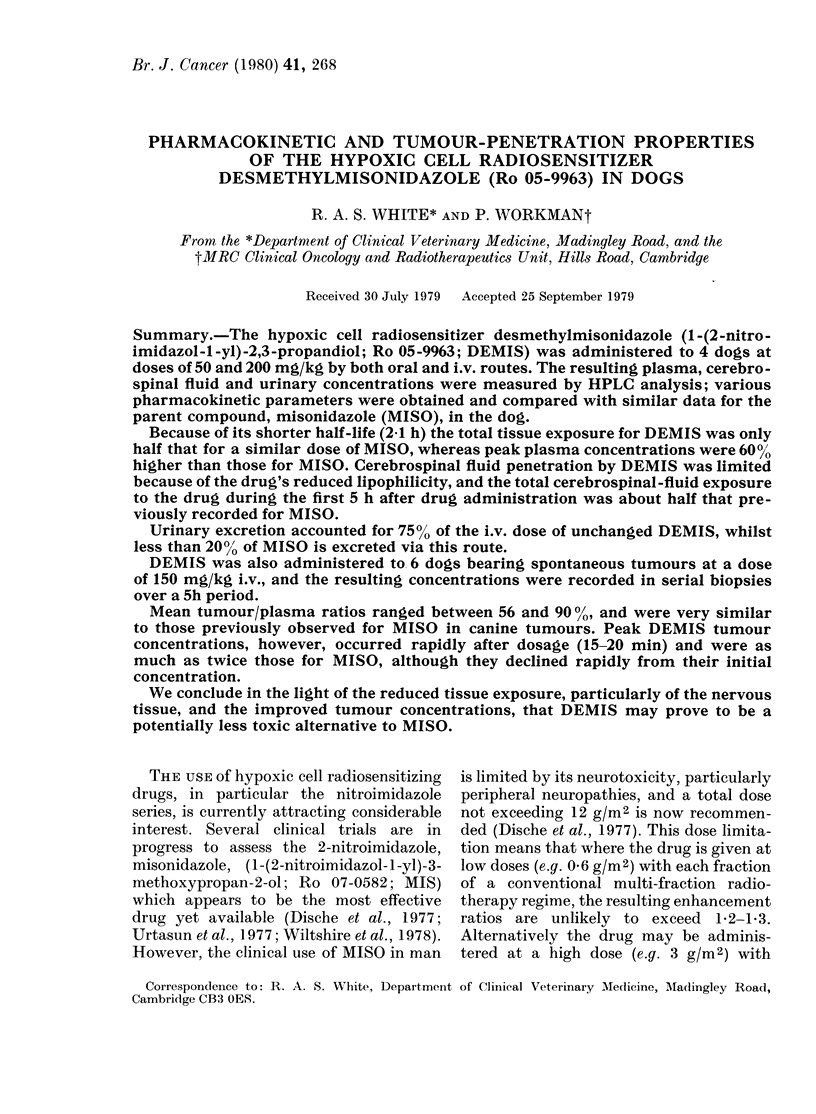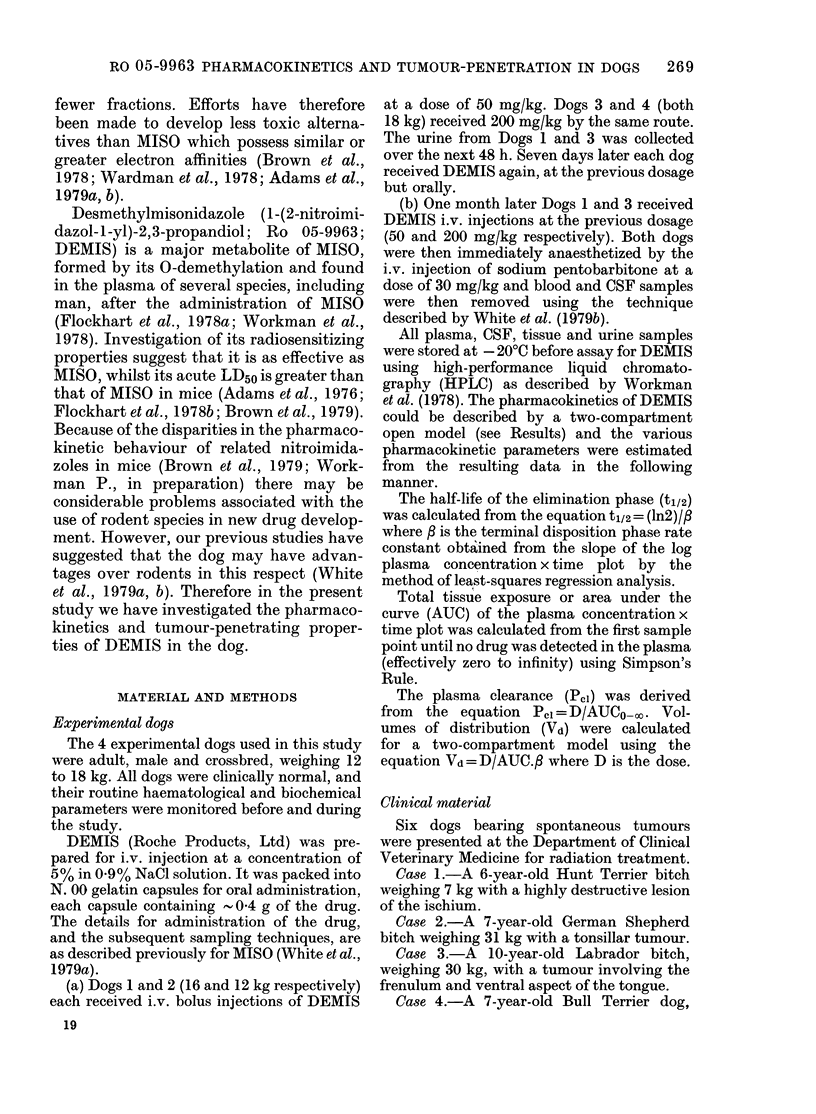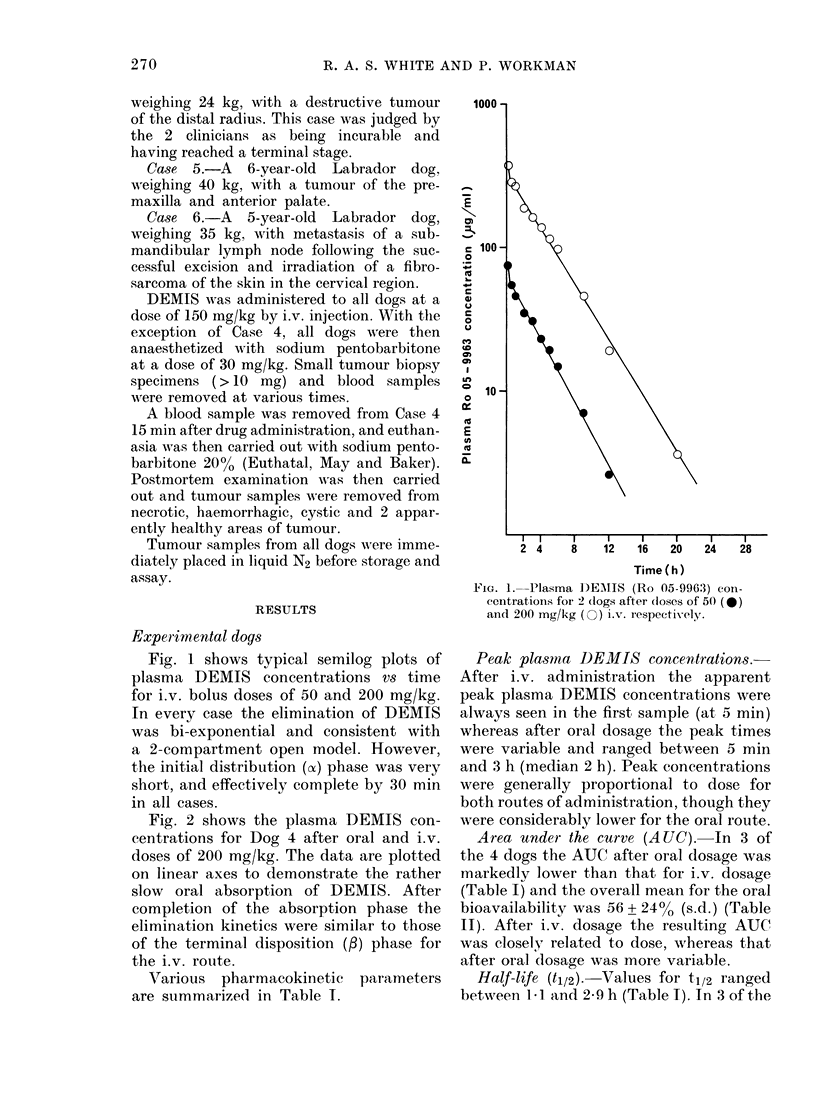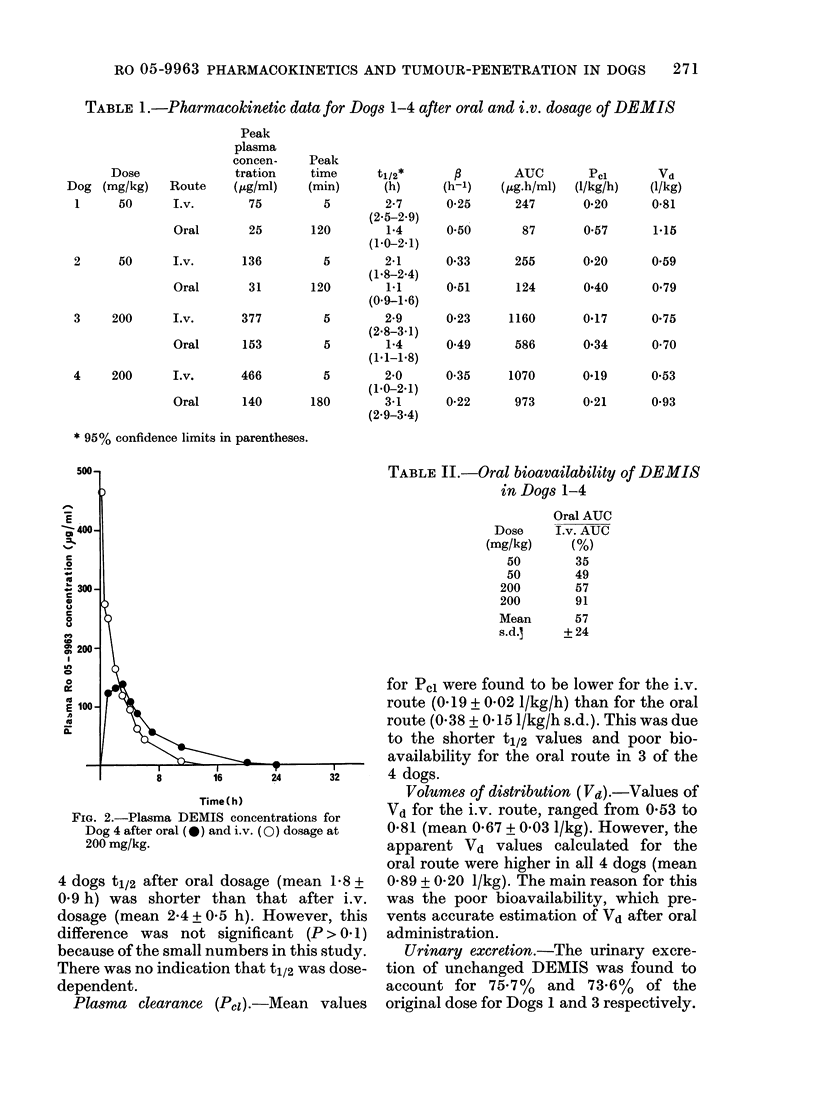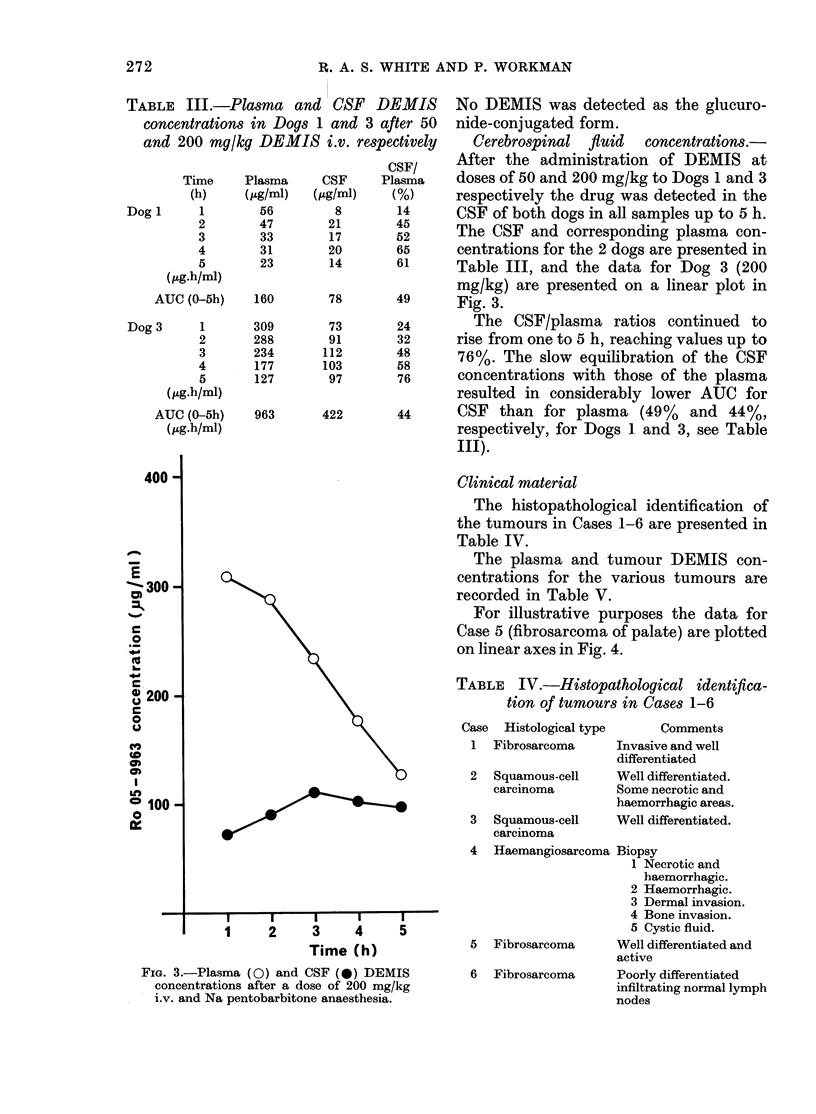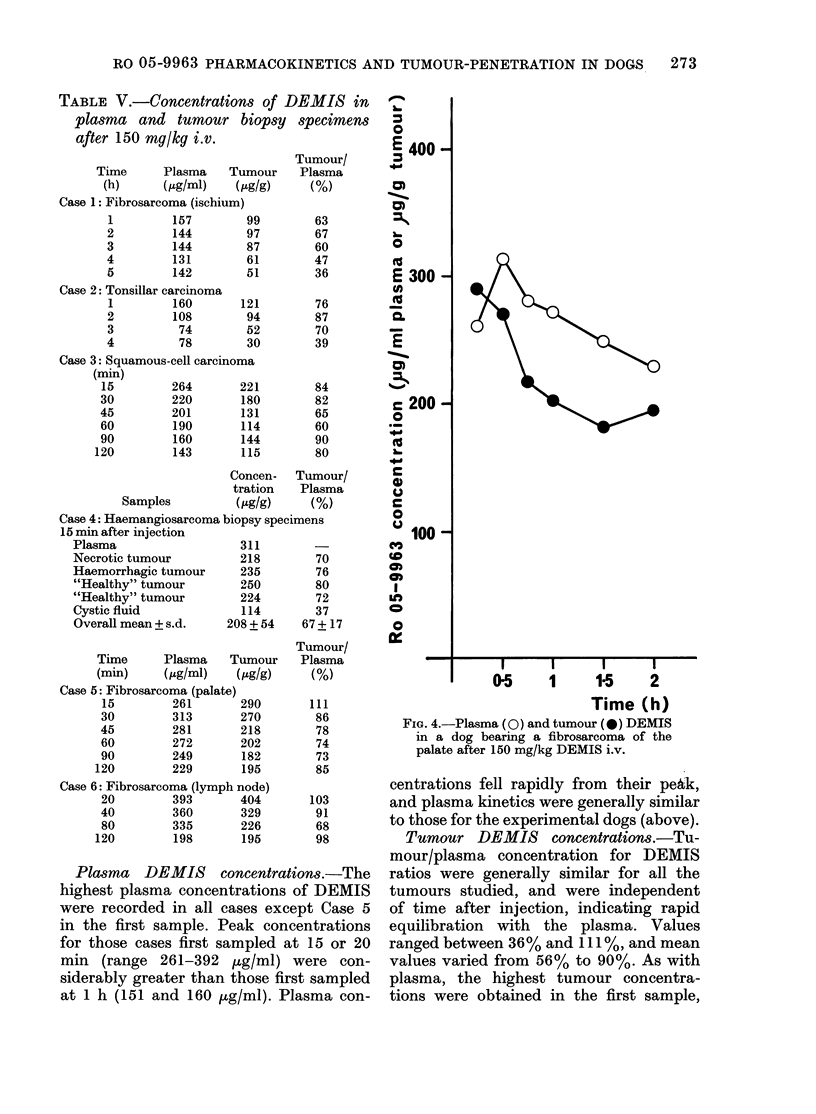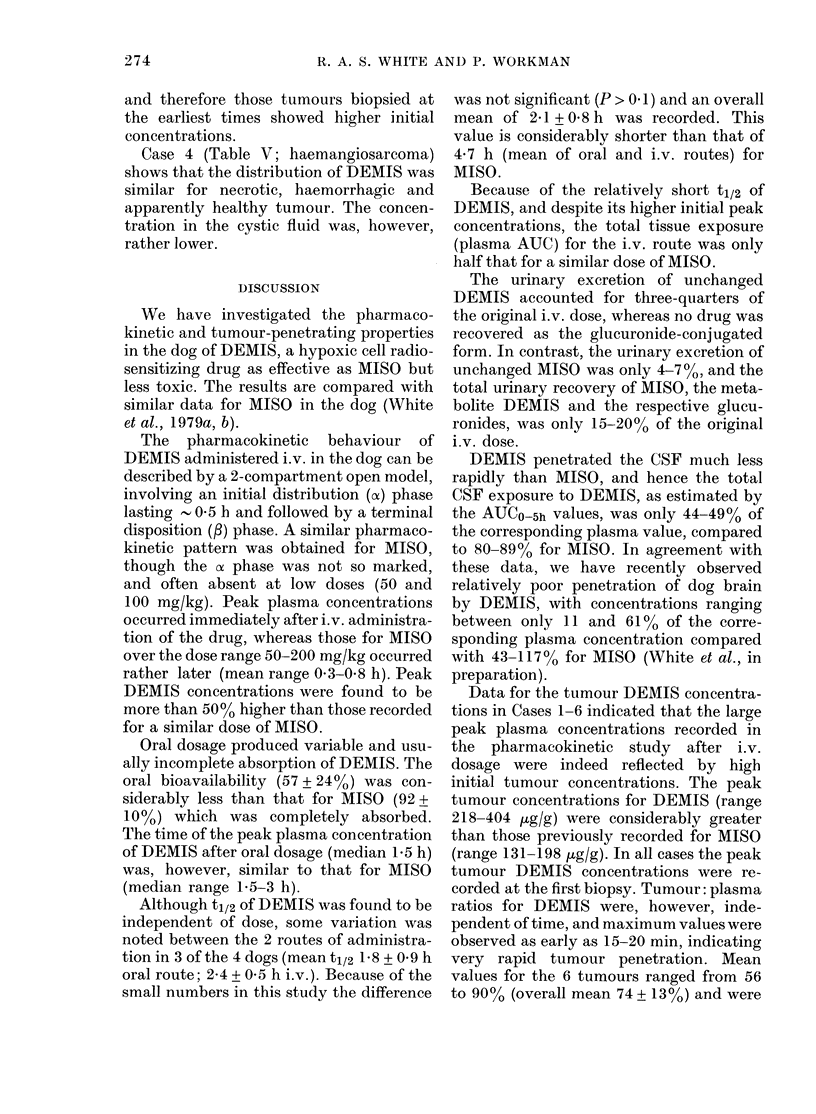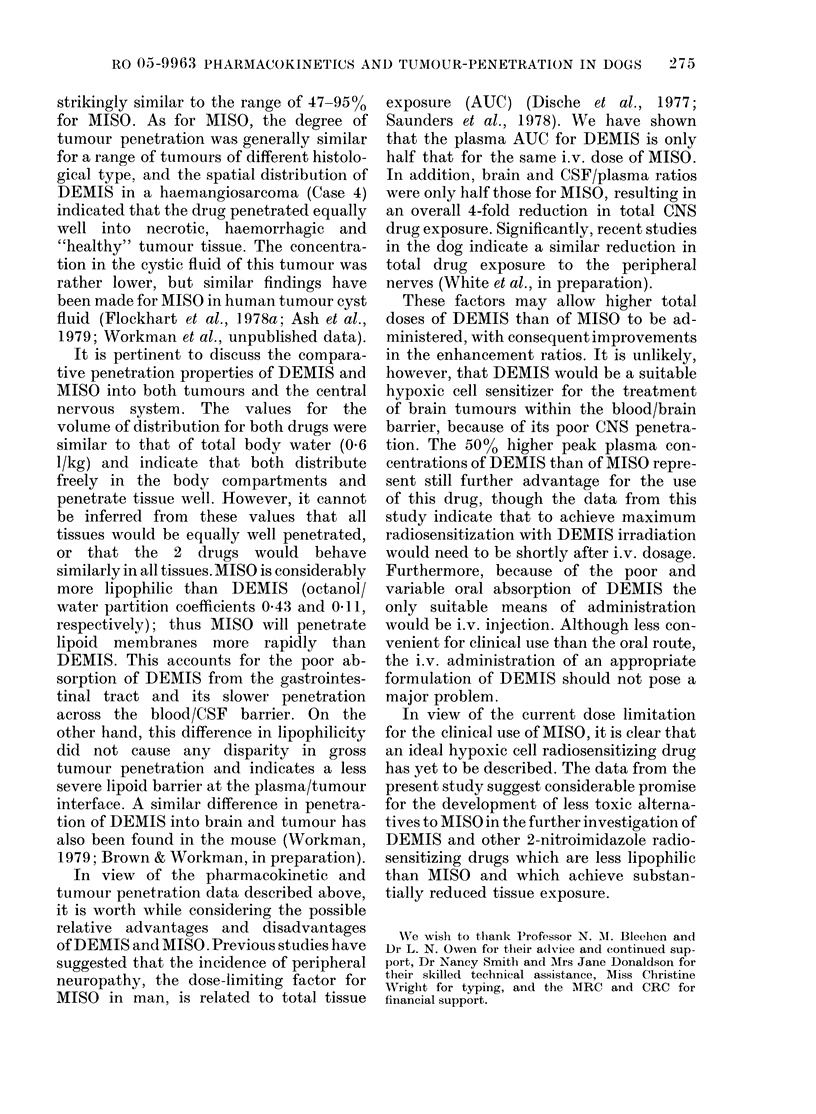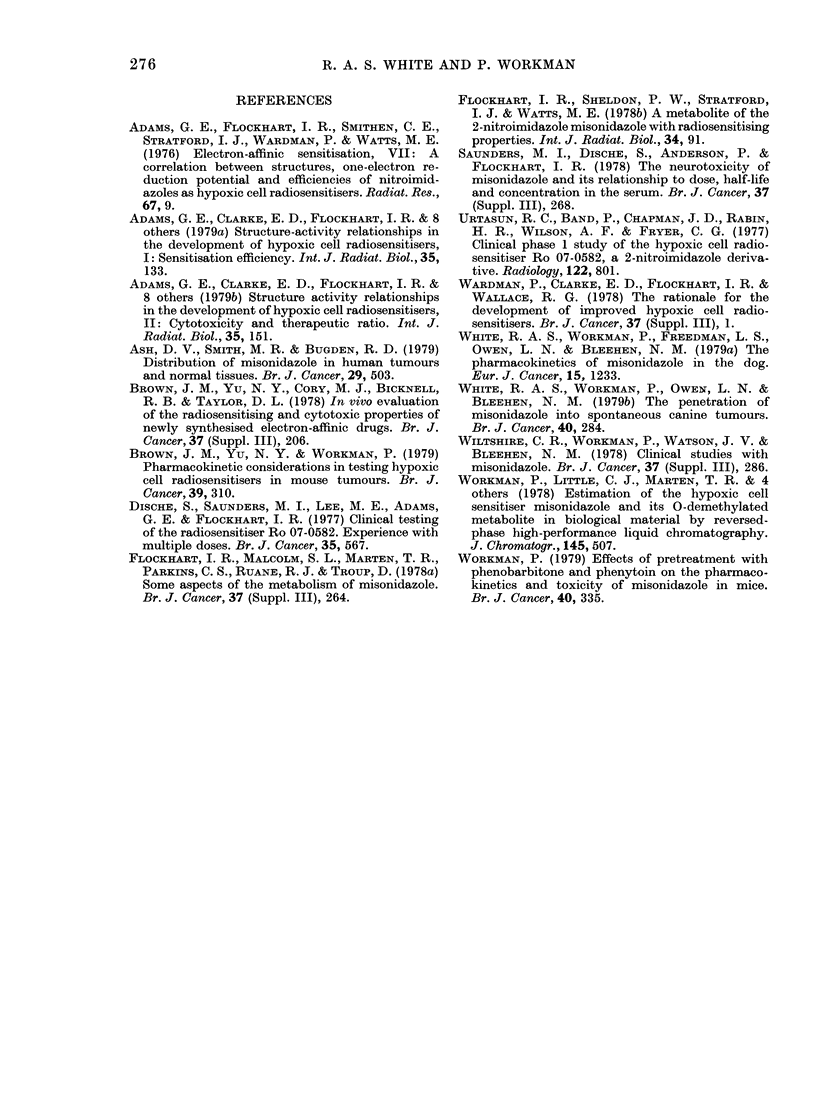# Pharmacokinetic and tumour-penetration properties of the hypoxic cell radiosensitizer desmethylmisonidazole (Ro 05-9963) in dogs

**DOI:** 10.1038/bjc.1980.39

**Published:** 1980-02

**Authors:** R. A. S. White, P. Workman

## Abstract

The hypoxic cell radiosensitizer desmethylmisonidazole (1-(2-nitroimidazol-1-yl)-2,3-propandiol; Ro 05-9963; DEMIS) was administered to 4 dogs at doses of 50 and 200 mg/kg by both oral and i.v. routes. The resulting plasma, cerebrospinal fluid and urinary concentrations were measured by HPLC analysis; various pharmacokinetic parameters were obtained and compared with similar data for the parent compound, misonidazole (MISO), in the dog.

Because of its shorter half-life (2·1 h) the total tissue exposure for DEMIS was only half that for a similar dose of MISO, whereas peak plasma concentrations were 60% higher than those for MISO. Cerebrospinal fluid penetration by DEMIS was limited because of the drug's reduced lipophilicity, and the total cerebrospinal-fluid exposure to the drug during the first 5 h after drug administration was about half that previously recorded for MISO.

Urinary excretion accounted for 75% of the i.v. dose of unchanged DEMIS, whilst less than 20% of MISO is excreted via this route.

DEMIS was also administered to 6 dogs bearing spontaneous tumours at a dose of 150 mg/kg i.v., and the resulting concentrations were recorded in serial biopsies over a 5h period.

Mean tumour/plasma ratios ranged between 56 and 90%, and were very similar to those previously observed for MISO in canine tumours. Peak DEMIS tumour concentrations, however, occurred rapidly after dosage (15-20 min) and were as much as twice those for MISO, although they declined rapidly from their initial concentration.

We conclude in the light of the reduced tissue exposure, particularly of the nervous tissue, and the improved tumour concentrations, that DEMIS may prove to be a potentially less toxic alternative to MISO.


*Br. J. Cancer* (1980) **41**, 268

### PHARMACOKINETIC AND TUMOUR-PENETRATION PROPERTIES OF THE HYPOXIC CELL RADIOSENSITIZER DESMETHYLMISONIDAZOLE (Ro 05-9963) IN DOGS

R. A. S. WHITE* AND P. WORKMAN†

*From the *Department of Clinical Veterinary Medicine, Madingley Road, and the †MRC Clinical Oncology and Radiotherapeutics Unit, Hills Road, Cambridge*

Received 30 July 1979 Accepted 25 September 1979

**Summary.—The hypoxic cell radiosensitizer desmethylmisonidazole (1-(2-nitro-imidazol-1-yl)-2,3-propandiol; Ro 05-9963; DEMIS) was administered to 4 dogs at doses of 50 and 200 mg/kg by both oral and i.v. routes. The resulting plasma, cerebrospinal fluid and urinary concentrations were measured by HPLC analysis; various pharmacokinetic parameters were obtained and compared with similar data for the parent compound, misonidazole (MISO), in the dog.**

**Because of its shorter half-life (2·1 h) the total tissue exposure for DEMIS was only half that for a similar dose of MISO, whereas peak plasma concentrations were 60% higher than those for MISO. Cerebrospinal fluid penetration by DEMIS was limited because of the drug's reduced lipophilicity, and the total cerebrospinal-fluid exposure to the drug during the first 5 h after drug administration was about half that previously recorded for MISO.**

**Urinary excretion accounted for 75% of the i.v. dose of unchanged DEMIS, whilst less than 20% of MISO is excreted via this route.**

**DEMIS was also administered to 6 dogs bearing spontaneous tumours at a dose of 150 mg/kg i.v., and the resulting concentrations were recorded in serial biopsies over a 5h period.**

**Mean tumour/plasma ratios ranged between 56 and 90%, and were very similar to those previously observed for MISO in canine tumours. Peak DEMIS tumour concentrations, however, occurred rapidly after dosage (15–20 min) and were as much as twice those for MISO, although they declined rapidly from their initial concentration.**

**We conclude in the light of the reduced tissue exposure, particularly of the nervous tissue, and the improved tumour concentrations, that DEMIS may prove to be a potentially less toxic alternative to MISO.**

THE USE of hypoxic cell radiosensitizing drugs, in particular the nitroimidazole series, is currently attracting considerable interest. Several clinical trials are in progress to assess the 2-nitroimidazole, misonidazole, (1-(2-nitroimidazol-1-yl)-3-methoxypropan-2-ol; Ro 07-0582; MIS) which appears to be the most effective drug yet available (Dische *et al.*, 1977; Urtasun *et al.*, 1977; Wiltshire *et al.*, 1978). However, the clinical use of MISO in man is limited by its neurotoxicity, particularly peripheral neuropathies, and a total dose not exceeding 12 $g/m^2$ is now recommended (Dische *et al.*, 1977). This dose limitation means that where the drug is given at low doses (*e.g.* 0·6 $g/m^2$) with each fraction of a conventional multi-fraction radiotherapy regime, the resulting enhancement ratios are unlikely to exceed 1·2–1·3. Alternatively the drug may be administered at a high dose (*e.g.* 3 $g/m^2$) with

Correspondence to: R. A. S. White, Department of Clinical Veterinary Medicine, Madingley Road, Cambridge CB3 0ES.

fewer fractions. Efforts have therefore been made to develop less toxic alternatives than MISO which possess similar or greater electron affinities (Brown *et al.*, 1978; Wardman *et al.*, 1978; Adams *et al.*, 1979*a*, *b*).

Desmethylmisonidazole (1-(2-nitroimidazol-1-yl)-2,3-propandiol; Ro 05-9963; DEMIS) is a major metabolite of MISO, formed by its O-demethylation and found in the plasma of several species, including man, after the administration of MISO (Flockhart *et al.*, 1978*a*; Workman *et al.*, 1978). Investigation of its radiosensitizing properties suggest that it is as effective as MISO, whilst its acute $LD_{50}$ is greater than that of MISO in mice (Adams *et al.*, 1976; Flockhart *et al.*, 1978*b*; Brown *et al.*, 1979). Because of the disparities in the pharmacokinetic behaviour of related nitroimidazoles in mice (Brown *et al.*, 1979; Workman P., in preparation) there may be considerable problems associated with the use of rodent species in new drug development. However, our previous studies have suggested that the dog may have advantages over rodents in this respect (White *et al.*, 1979*a*, *b*). Therefore in the present study we have investigated the pharmacokinetics and tumour-penetrating properties of DEMIS in the dog.

### MATERIAL AND METHODS

### *Experimental dogs*

The 4 experimental dogs used in this study were adult, male and crossbred, weighing 12 to 18 kg. All dogs were clinically normal, and their routine haematological and biochemical parameters were monitored before and during the study.

DEMIS (Roche Products, Ltd) was prepared for i.v. injection at a concentration of 5% in 0·9% NaCl solution. It was packed into N. 00 gelatin capsules for oral administration, each capsule containing ~0·4 g of the drug. The details for administration of the drug, and the subsequent sampling techniques, are as described previously for MISO (White *et al.*, 1979*a*).

(a) Dogs 1 and 2 (16 and 12 kg respectively) each received i.v. bolus injections of DEMIS at a dose of 50 mg/kg. Dogs 3 and 4 (both 18 kg) received 200 mg/kg by the same route. The urine from Dogs 1 and 3 was collected over the next 48 h. Seven days later each dog received DEMIS again, at the previous dosage but orally.

(b) One month later Dogs 1 and 3 received DEMIS i.v. injections at the previous dosage (50 and 200 mg/kg respectively). Both dogs were then immediately anaesthetized by the i.v. injection of sodium pentobarbitone at a dose of 30 mg/kg and blood and CSF samples were then removed using the technique described by White *et al.* (1979*b*).

All plasma, CSF, tissue and urine samples were stored at −20°C before assay for DEMIS using high-performance liquid chromatography (HPLC) as described by Workman *et al.* (1978). The pharmacokinetics of DEMIS could be described by a two-compartment open model (see Results) and the various pharmacokinetic parameters were estimated from the resulting data in the following manner.

The half-life of the elimination phase ($t_{1/2}$) was calculated from the equation $t_{1/2} = (\ln 2)/\beta$ where $\beta$ is the terminal disposition phase rate constant obtained from the slope of the log plasma concentration × time plot by the method of least-squares regression analysis.

Total tissue exposure or area under the curve (AUC) of the plasma concentration × time plot was calculated from the first sample point until no drug was detected in the plasma (effectively zero to infinity) using Simpson's Rule.

The plasma clearance ($P_{cl}$) was derived from the equation $P_{cl} = D/AUC_{0-\infty}$. Volumes of distribution ($V_d$) were calculated for a two-compartment model using the equation $V_d = D/AUC.\beta$ where D is the dose.

### *Clinical material*

Six dogs bearing spontaneous tumours were presented at the Department of Clinical Veterinary Medicine for radiation treatment.

*Case* 1.—A 6-year-old Hunt Terrier bitch weighing 7 kg with a highly destructive lesion of the ischium.

*Case* 2.—A 7-year-old German Shepherd bitch weighing 31 kg with a tonsillar tumour.

*Case* 3.—A 10-year-old Labrador bitch, weighing 30 kg, with a tumour involving the frenulum and ventral aspect of the tongue.

*Case* 4.—A 7-year-old Bull Terrier dog, weighing 24 kg, with a destructive tumour of the distal radius. This case was judged by the 2 clinicians as being incurable and having reached a terminal stage.

*Case* 5.—A 6-year-old Labrador dog, weighing 40 kg, with a tumour of the premaxilla and anterior palate.

*Case* 6.—A 5-year-old Labrador dog, weighing 35 kg, with metastasis of a submandibular lymph node following the successful excision and irradiation of a fibrosarcoma of the skin in the cervical region.

DEMIS was administered to all dogs at a dose of 150 mg/kg by i.v. injection. With the exception of Case 4, all dogs were then anaesthetized with sodium pentobarbitone at a dose of 30 mg/kg. Small tumour biopsy specimens ($>10$ mg) and blood samples were removed at various times.

A blood sample was removed from Case 4 15 min after drug administration, and euthanasia was then carried out with sodium pentobarbitone 20% (Euthatal, May and Baker). Postmortem examination was then carried out and tumour samples were removed from necrotic, haemorrhagic, cystic and 2 apparently healthy areas of tumour.

Tumour samples from all dogs were immediately placed in liquid $N_2$ before storage and assay.

FIG. 1.—Plasma DEMIS (Ro 05-9963) concentrations for 2 dogs after doses of 50 (●) and 200 mg/kg (○) i.v. respectively.

### RESULTS

### *Experimental dogs*

Fig. 1 shows typical semilog plots of plasma DEMIS concentrations *vs* time for i.v. bolus doses of 50 and 200 mg/kg. In every case the elimination of DEMIS was bi-exponential and consistent with a 2-compartment open model. However, the initial distribution ($\alpha$) phase was very short, and effectively complete by 30 min in all cases.

Fig. 2 shows the plasma DEMIS concentrations for Dog 4 after oral and i.v. doses of 200 mg/kg. The data are plotted on linear axes to demonstrate the rather slow oral absorption of DEMIS. After completion of the absorption phase the elimination kinetics were similar to those of the terminal disposition ($\beta$) phase for the i.v. route.

Various pharmacokinetic parameters are summarized in Table I.

*Peak plasma DEMIS concentrations.*—After i.v. administration the apparent peak plasma DEMIS concentrations were always seen in the first sample (at 5 min) whereas after oral dosage the peak times were variable and ranged between 5 min and 3 h (median 2 h). Peak concentrations were generally proportional to dose for both routes of administration, though they were considerably lower for the oral route.

*Area under the curve* (*AUC*).—In 3 of the 4 dogs the AUC after oral dosage was markedly lower than that for i.v. dosage (Table I) and the overall mean for the oral bioavailability was $56 \pm 24\%$ (s.d.) (Table II). After i.v. dosage the resulting AUC was closely related to dose, whereas that after oral dosage was more variable.

*Half-life* ($t_{1/2}$).—Values for $t_{1/2}$ ranged between 1·1 and 2·9 h (Table I). In 3 of the

TABLE I.—*Pharmacokinetic data for Dogs 1–4 after oral and i.v. dosage of DEMIS*

| Dog | Dose (mg/kg) | Route | Peak plasma concentration ($\mu$g/ml) | Peak time (min) | $t_{1/2}$* (h) | $\beta$ ($h^{-1}$) | AUC ($\mu$g.h/ml) | $P_{cl}$ (l/kg/h) | $V_d$ (l/kg) |
|---|---|---|---|---|---|---|---|---|---|
| 1 | 50 | I.v. | 75 | 5 | 2·7 (2·5–2·9) | 0·25 | 247 | 0·20 | 0·81 |
| | | Oral | 25 | 120 | 1·4 (1·0–2·1) | 0·50 | 87 | 0·57 | 1·15 |
| 2 | 50 | I.v. | 136 | 5 | 2·1 (1·8–2·4) | 0·33 | 255 | 0·20 | 0·59 |
| | | Oral | 31 | 120 | 1·1 (0·9–1·6) | 0·51 | 124 | 0·40 | 0·79 |
| 3 | 200 | I.v. | 377 | 5 | 2·9 (2·8–3·1) | 0·23 | 1160 | 0·17 | 0·75 |
| | | Oral | 153 | 5 | 1·4 (1·1–1·8) | 0·49 | 586 | 0·34 | 0·70 |
| 4 | 200 | I.v. | 466 | 5 | 2·0 (1·0–2·1) | 0·35 | 1070 | 0·19 | 0·53 |
| | | Oral | 140 | 180 | 3·1 (2·9–3·4) | 0·22 | 973 | 0·21 | 0·93 |

* 95% confidence limits in parentheses.

FIG. 2.—Plasma DEMIS concentrations for Dog 4 after oral (●) and i.v. (○) dosage at 200 mg/kg.

4 dogs $t_{1/2}$ after oral dosage (mean 1·8 ± 0·9 h) was shorter than that after i.v. dosage (mean 2·4 ± 0·5 h). However, this difference was not significant ($P > 0{\cdot}1$) because of the small numbers in this study. There was no indication that $t_{1/2}$ was dose-dependent.

*Plasma clearance* (*$P_{cl}$*).—Mean values for $P_{cl}$ were found to be lower for the i.v. route (0·19 ± 0·02 l/kg/h) than for the oral route (0·38 ± 0·15 l/kg/h s.d.). This was due to the shorter $t_{1/2}$ values and poor bioavailability for the oral route in 3 of the 4 dogs.

TABLE II.—*Oral bioavailability of DEMIS in Dogs* 1–4

| Dose (mg/kg) | Oral AUC / I.v. AUC (%) |
|---|---|
| 50 | 35 |
| 50 | 49 |
| 200 | 57 |
| 200 | 91 |
| Mean | 57 |
| s.d. | ± 24 |

*Volumes of distribution* (*$V_d$*).—Values of $V_d$ for the i.v. route, ranged from 0·53 to 0·81 (mean 0·67 ± 0·03 l/kg). However, the apparent $V_d$ values calculated for the oral route were higher in all 4 dogs (mean 0·89 ± 0·20 l/kg). The main reason for this was the poor bioavailability, which prevents accurate estimation of $V_d$ after oral administration.

*Urinary excretion.*—The urinary excretion of unchanged DEMIS was found to account for 75·7% and 73·6% of the original dose for Dogs 1 and 3 respectively.

TABLE III.—*Plasma and CSF DEMIS concentrations in Dogs* 1 *and* 3 *after* 50 *and* 200 *mg/kg DEMIS i.v. respectively*

| | Time (h) | Plasma (μg/ml) | CSF (μg/ml) | CSF/ Plasma (%) |
|---|---|---|---|---|
| Dog 1 | 1 | 56 | 8 | 14 |
| | 2 | 47 | 21 | 45 |
| | 3 | 33 | 17 | 52 |
| | 4 | 31 | 20 | 65 |
| | 5 | 23 | 14 | 61 |
| | (μg.h/ml) | | | |
| | AUC (0–5h) | 160 | 78 | 49 |
| Dog 3 | 1 | 309 | 73 | 24 |
| | 2 | 288 | 91 | 32 |
| | 3 | 234 | 112 | 48 |
| | 4 | 177 | 103 | 58 |
| | 5 | 127 | 97 | 76 |
| | (μg.h/ml) | | | |
| | AUC (0–5h) (μg.h/ml) | 963 | 422 | 44 |

FIG. 3.—Plasma (○) and CSF (●) DEMIS concentrations after a dose of 200 mg/kg i.v. and Na pentobarbitone anaesthesia.

No DEMIS was detected as the glucuronide-conjugated form.

*Cerebrospinal fluid concentrations.*—After the administration of DEMIS at doses of 50 and 200 mg/kg to Dogs 1 and 3 respectively the drug was detected in the CSF of both dogs in all samples up to 5 h. The CSF and corresponding plasma concentrations for the 2 dogs are presented in Table III, and the data for Dog 3 (200 mg/kg) are presented on a linear plot in Fig. 3.

The CSF/plasma ratios continued to rise from one to 5 h, reaching values up to 76%. The slow equilibration of the CSF concentrations with those of the plasma resulted in considerably lower AUC for CSF than for plasma (49% and 44%, respectively, for Dogs 1 and 3, see Table III).

### *Clinical material*

The histopathological identification of the tumours in Cases 1–6 are presented in Table IV.

The plasma and tumour DEMIS concentrations for the various tumours are recorded in Table V.

For illustrative purposes the data for Case 5 (fibrosarcoma of palate) are plotted on linear axes in Fig. 4.

TABLE IV.—*Histopathological identification of tumours in Cases* 1–6

| Case | Histological type | Comments |
|---|---|---|
| 1 | Fibrosarcoma | Invasive and well differentiated |
| 2 | Squamous-cell carcinoma | Well differentiated. Some necrotic and haemorrhagic areas. |
| 3 | Squamous-cell carcinoma | Well differentiated. |
| 4 | Haemangiosarcoma | Biopsy<br>1 Necrotic and haemorrhagic.<br>2 Haemorrhagic.<br>3 Dermal invasion.<br>4 Bone invasion.<br>5 Cystic fluid. |
| 5 | Fibrosarcoma | Well differentiated and active |
| 6 | Fibrosarcoma | Poorly differentiated infiltrating normal lymph nodes |

TABLE V.—*Concentrations of DEMIS in plasma and tumour biopsy specimens after* 150 *mg/kg i.v.*

| Time (h) | Plasma (μg/ml) | Tumour (μg/g) | Tumour/ Plasma (%) |
|---|---|---|---|
| Case 1: Fibrosarcoma (ischium) | | | |
| 1 | 157 | 99 | 63 |
| 2 | 144 | 97 | 67 |
| 3 | 144 | 87 | 60 |
| 4 | 131 | 61 | 47 |
| 5 | 142 | 51 | 36 |
| Case 2: Tonsillar carcinoma | | | |
| 1 | 160 | 121 | 76 |
| 2 | 108 | 94 | 87 |
| 3 | 74 | 52 | 70 |
| 4 | 78 | 30 | 39 |
| Case 3: Squamous-cell carcinoma (min) | | | |
| 15 | 264 | 221 | 84 |
| 30 | 220 | 180 | 82 |
| 45 | 201 | 131 | 65 |
| 60 | 190 | 114 | 60 |
| 90 | 160 | 144 | 90 |
| 120 | 143 | 115 | 80 |

| Samples | Concentration (μg/g) | Tumour/ Plasma (%) |
|---|---|---|
| Case 4: Haemangiosarcoma biopsy specimens 15 min after injection | | |
| Plasma | 311 | — |
| Necrotic tumour | 218 | 70 |
| Haemorrhagic tumour | 235 | 76 |
| "Healthy" tumour | 250 | 80 |
| "Healthy" tumour | 224 | 72 |
| Cystic fluid | 114 | 37 |
| Overall mean ± s.d. | 208 ± 54 | 67 ± 17 |

| Time (min) | Plasma (μg/ml) | Tumour (μg/g) | Tumour/ Plasma (%) |
|---|---|---|---|
| Case 5: Fibrosarcoma (palate) | | | |
| 15 | 261 | 290 | 111 |
| 30 | 313 | 270 | 86 |
| 45 | 281 | 218 | 78 |
| 60 | 272 | 202 | 74 |
| 90 | 249 | 182 | 73 |
| 120 | 229 | 195 | 85 |
| Case 6: Fibrosarcoma (lymph node) | | | |
| 20 | 393 | 404 | 103 |
| 40 | 360 | 329 | 91 |
| 80 | 335 | 226 | 68 |
| 120 | 198 | 195 | 98 |

*Plasma DEMIS concentrations*.—The highest plasma concentrations of DEMIS were recorded in all cases except Case 5 in the first sample. Peak concentrations for those cases first sampled at 15 or 20 min (range 261–392 μg/ml) were considerably greater than those first sampled at 1 h (151 and 160 μg/ml). Plasma con-

FIG. 4.—Plasma (○) and tumour (●) DEMIS in a dog bearing a fibrosarcoma of the palate after 150 mg/kg DEMIS i.v.

centrations fell rapidly from their peak, and plasma kinetics were generally similar to those for the experimental dogs (above).

*Tumour DEMIS concentrations*.—Tumour/plasma concentration for DEMIS ratios were generally similar for all the tumours studied, and were independent of time after injection, indicating rapid equilibration with the plasma. Values ranged between 36% and 111%, and mean values varied from 56% to 90%. As with plasma, the highest tumour concentrations were obtained in the first sample, and therefore those tumours biopsied at the earliest times showed higher initial concentrations.

Case 4 (Table V; haemangiosarcoma) shows that the distribution of DEMIS was similar for necrotic, haemorrhagic and apparently healthy tumour. The concentration in the cystic fluid was, however, rather lower.

### DISCUSSION

We have investigated the pharmacokinetic and tumour-penetrating properties in the dog of DEMIS, a hypoxic cell radiosensitizing drug as effective as MISO but less toxic. The results are compared with similar data for MISO in the dog (White *et al.*, 1979*a*, *b*).

The pharmacokinetic behaviour of DEMIS administered i.v. in the dog can be described by a 2-compartment open model, involving an initial distribution ($\alpha$) phase lasting $\sim 0{\cdot}5$ h and followed by a terminal disposition ($\beta$) phase. A similar pharmacokinetic pattern was obtained for MISO, though the $\alpha$ phase was not so marked, and often absent at low doses (50 and 100 mg/kg). Peak plasma concentrations occurred immediately after i.v. administration of the drug, whereas those for MISO over the dose range 50–200 mg/kg occurred rather later (mean range 0·3–0·8 h). Peak DEMIS concentrations were found to be more than 50% higher than those recorded for a similar dose of MISO.

Oral dosage produced variable and usually incomplete absorption of DEMIS. The oral bioavailability ($57 \pm 24\%$) was considerably less than that for MISO ($92 \pm 10\%$) which was completely absorbed. The time of the peak plasma concentration of DEMIS after oral dosage (median 1·5 h) was, however, similar to that for MISO (median range 1·5–3 h).

Although $t_{1/2}$ of DEMIS was found to be independent of dose, some variation was noted between the 2 routes of administration in 3 of the 4 dogs (mean $t_{1/2}$ $1{\cdot}8 \pm 0{\cdot}9$ h oral route; $2{\cdot}4 \pm 0{\cdot}5$ h i.v.). Because of the small numbers in this study the difference was not significant ($P > 0{\cdot}1$) and an overall mean of $2{\cdot}1 \pm 0{\cdot}8$ h was recorded. This value is considerably shorter than that of 4·7 h (mean of oral and i.v. routes) for MISO.

Because of the relatively short $t_{1/2}$ of DEMIS, and despite its higher initial peak concentrations, the total tissue exposure (plasma AUC) for the i.v. route was only half that for a similar dose of MISO.

The urinary excretion of unchanged DEMIS accounted for three-quarters of the original i.v. dose, whereas no drug was recovered as the glucuronide-conjugated form. In contrast, the urinary excretion of unchanged MISO was only 4–7%, and the total urinary recovery of MISO, the metabolite DEMIS and the respective glucuronides, was only 15–20% of the original i.v. dose.

DEMIS penetrated the CSF much less rapidly than MISO, and hence the total CSF exposure to DEMIS, as estimated by the $AUC_{0-5h}$ values, was only 44–49% of the corresponding plasma value, compared to 80–89% for MISO. In agreement with these data, we have recently observed relatively poor penetration of dog brain by DEMIS, with concentrations ranging between only 11 and 61% of the corresponding plasma concentration compared with 43–117% for MISO (White *et al.*, in preparation).

Data for the tumour DEMIS concentrations in Cases 1–6 indicated that the large peak plasma concentrations recorded in the pharmacokinetic study after i.v. dosage were indeed reflected by high initial tumour concentrations. The peak tumour concentrations for DEMIS (range 218–404 $\mu$g/g) were considerably greater than those previously recorded for MISO (range 131–198 $\mu$g/g). In all cases the peak tumour DEMIS concentrations were recorded at the first biopsy. Tumour: plasma ratios for DEMIS were, however, independent of time, and maximum values were observed as early as 15–20 min, indicating very rapid tumour penetration. Mean values for the 6 tumours ranged from 56 to 90% (overall mean $74 \pm 13\%$) and were strikingly similar to the range of 47–95% for MISO. As for MISO, the degree of tumour penetration was generally similar for a range of tumours of different histological type, and the spatial distribution of DEMIS in a haemangiosarcoma (Case 4) indicated that the drug penetrated equally well into necrotic, haemorrhagic and "healthy" tumour tissue. The concentration in the cystic fluid of this tumour was rather lower, but similar findings have been made for MISO in human tumour cyst fluid (Flockhart *et al.*, 1978*a*; Ash *et al.*, 1979; Workman *et al.*, unpublished data).

It is pertinent to discuss the comparative penetration properties of DEMIS and MISO into both tumours and the central nervous system. The values for the volume of distribution for both drugs were similar to that of total body water (0·6 l/kg) and indicate that both distribute freely in the body compartments and penetrate tissue well. However, it cannot be inferred from these values that all tissues would be equally well penetrated, or that the 2 drugs would behave similarly in all tissues. MISO is considerably more lipophilic than DEMIS (octanol/water partition coefficients 0·43 and 0·11, respectively); thus MISO will penetrate lipoid membranes more rapidly than DEMIS. This accounts for the poor absorption of DEMIS from the gastrointestinal tract and its slower penetration across the blood/CSF barrier. On the other hand, this difference in lipophilicity did not cause any disparity in gross tumour penetration and indicates a less severe lipoid barrier at the plasma/tumour interface. A similar difference in penetration of DEMIS into brain and tumour has also been found in the mouse (Workman, 1979; Brown & Workman, in preparation).

In view of the pharmacokinetic and tumour penetration data described above, it is worth while considering the possible relative advantages and disadvantages of DEMIS and MISO. Previous studies have suggested that the incidence of peripheral neuropathy, the dose-limiting factor for MISO in man, is related to total tissue exposure (AUC) (Dische *et al.*, 1977; Saunders *et al.*, 1978). We have shown that the plasma AUC for DEMIS is only half that for the same i.v. dose of MISO. In addition, brain and CSF/plasma ratios were only half those for MISO, resulting in an overall 4-fold reduction in total CNS drug exposure. Significantly, recent studies in the dog indicate a similar reduction in total drug exposure to the peripheral nerves (White *et al.*, in preparation).

These factors may allow higher total doses of DEMIS than of MISO to be administered, with consequent improvements in the enhancement ratios. It is unlikely, however, that DEMIS would be a suitable hypoxic cell sensitizer for the treatment of brain tumours within the blood/brain barrier, because of its poor CNS penetration. The 50% higher peak plasma concentrations of DEMIS than of MISO represent still further advantage for the use of this drug, though the data from this study indicate that to achieve maximum radiosensitization with DEMIS irradiation would need to be shortly after i.v. dosage. Furthermore, because of the poor and variable oral absorption of DEMIS the only suitable means of administration would be i.v. injection. Although less convenient for clinical use than the oral route, the i.v. administration of an appropriate formulation of DEMIS should not pose a major problem.

In view of the current dose limitation for the clinical use of MISO, it is clear that an ideal hypoxic cell radiosensitizing drug has yet to be described. The data from the present study suggest considerable promise for the development of less toxic alternatives to MISO in the further investigation of DEMIS and other 2-nitroimidazole radiosensitizing drugs which are less lipophilic than MISO and which achieve substantially reduced tissue exposure.

We wish to thank Professor N. M. Bleehen and Dr L. N. Owen for their advice and continued support, Dr Nancy Smith and Mrs Jane Donaldson for their skilled technical assistance, Miss Christine Wright for typing, and the MRC and CRC for financial support.

### REFERENCES

ADAMS, G. E., FLOCKHART, I. R., SMITHEN, C. E., STRATFORD, I. J., WARDMAN, P. & WATTS, M. E. (1976) Electron-affinic sensitisation, VII: A correlation between structures, one-electron reduction potential and efficiencies of nitroimidazoles as hypoxic cell radiosensitisers. *Radiat. Res.*, **67**, 9.

ADAMS, G. E., CLARKE, E. D., FLOCKHART, I. R. & 8 others (1979*a*) Structure-activity relationships in the development of hypoxic cell radiosensitisers, I: Sensitisation efficiency. *Int. J. Radiat. Biol.*, **35**, 133.

ADAMS, G. E., CLARKE, E. D., FLOCKHART, I. R. & 8 others (1979*b*) Structure activity relationships in the development of hypoxic cell radiosensitisers, II: Cytotoxicity and therapeutic ratio. *Int. J. Radiat. Biol.*, **35**, 151.

ASH, D. V., SMITH, M. R. & BUGDEN, R. D. (1979) Distribution of misonidazole in human tumours and normal tissues. *Br. J. Cancer*, **29**, 503.

BROWN, J. M., YU, N. Y., CORY, M. J., BICKNELL, R. B. & TAYLOR, D. L. (1978) *In vivo* evaluation of the radiosensitising and cytotoxic properties of newly synthesised electron-affinic drugs. *Br. J. Cancer*, **37** (Suppl. III), 206.

BROWN, J. M., YU, N. Y. & WORKMAN, P. (1979) Pharmacokinetic considerations in testing hypoxic cell radiosensitisers in mouse tumours. *Br. J. Cancer*, **39**, 310.

DISCHE, S., SAUNDERS, M. I., LEE, M. E., ADAMS, G. E. & FLOCKHART, I. R. (1977) Clinical testing of the radiosensitiser Ro 07-0582. Experience with multiple doses. *Br. J. Cancer*, **35**, 567.

FLOCKHART, I. R., MALCOLM, S. L., MARTEN, T. R., PARKINS, C. S., RUANE, R. J. & TROUP, D. (1978*a*) Some aspects of the metabolism of misonidazole. *Br. J. Cancer*, **37** (Suppl. III), 264.

FLOCKHART, I. R., SHELDON, P. W., STRATFORD, I. J. & WATTS, M. E. (1978*b*) A metabolite of the 2-nitroimidazole misonidazole with radiosensitising properties. *Int. J. Radiat. Biol.*, **34**, 91.

SAUNDERS, M. I., DISCHE, S., ANDERSON, P. & FLOCKHART, I. R. (1978) The neurotoxicity of misonidazole and its relationship to dose, half-life and concentration in the serum. *Br. J. Cancer*, **37** (Suppl. III), 268.

URTASUN, R. C., BAND, P., CHAPMAN, J. D., RABIN, H. R., WILSON, A. F. & FRYER, C. G. (1977) Clinical phase 1 study of the hypoxic cell radiosensitiser Ro 07-0582, a 2-nitroimidazole derivative. *Radiology*, **122**, 801.

WARDMAN, P., CLARKE, E. D., FLOCKHART, I. R. & WALLACE, R. G. (1978) The rationale for the development of improved hypoxic cell radiosensitisers. *Br. J. Cancer*, **37** (Suppl. III), 1.

WHITE, R. A. S., WORKMAN, P., FREEDMAN, L. S., OWEN, L. N. & BLEEHEN, N. M. (1979*a*) The pharmacokinetics of misonidazole in the dog. *Eur. J. Cancer*, **15**, 1233.

WHITE, R. A. S., WORKMAN, P., OWEN, L. N. & BLEEHEN, N. M. (1979*b*) The penetration of misonidazole into spontaneous canine tumours. *Br. J. Cancer*, **40**, 284.

WILTSHIRE, C. R., WORKMAN, P., WATSON, J. V. & BLEEHEN, N. M. (1978) Clinical studies with misonidazole. *Br. J. Cancer*, **37** (Suppl. III), 286.

WORKMAN, P., LITTLE, C. J., MARTEN, T. R. & 4 others (1978) Estimation of the hypoxic cell sensitiser misonidazole and its O-demethylated metabolite in biological material by reversed-phase high-performance liquid chromatography. *J. Chromatogr.*, **145**, 507.

WORKMAN, P. (1979) Effects of pretreatment with phenobarbitone and phenytoin on the pharmacokinetics and toxicity of misonidazole in mice. *Br. J. Cancer*, **40**, 335.